# Secreted frizzled-related protein 1 overexpression in gastric cancer: Relationship with radiological findings of dual-energy spectral CT and PET-CT

**DOI:** 10.1038/srep42020

**Published:** 2017-02-07

**Authors:** Huimin Lin, Guoyuan Yang, Bei Ding, Miao Zhang, Mingjun Zhang, Fuhua Yan, Ying Qu, Huan Zhang

**Affiliations:** 1 Department of Radiology, Ruijin Hospital, Shanghai Jiaotong University School of Medicine, Shanghai 200025, People’s Republic of China; 2 Department of Neurology, Ruijin Hospital, Shanghai Jiaotong University School of Medicine, Shanghai 200025, People’s Republic of China; 3 Department of Nuclear Medicine, Ruijin Hospital, Shanghai Jiaotong University School of Medicine, Shanghai 200025, People’s Republic of China; 4 Laboratory Animal Research Center, Ruijin Hospital, Shanghai Jiaotong University School of Medicine, Shanghai 200025, People’s Republic of China; 5 Cedars-Sinai medical center, 8700 beverly Blvd, Los Angeles, Ca90048, USA

## Abstract

We explored the role of secreted frizzled-related protein 1 (sFRP1) overexpression in gastric cancer and its relationship with radiological findings from dual-energy spectral CT(DEsCT) and positron emission tomography/computed tomography (PET/CT). We established mouse metastatic models using the SGC-7901/sFRP1 gastric cancer cell line. A control group was established using the SGC-7901/vector cell line. The models were then scanned with dual-energy spectral CT and PET-CT. Subsequent analysis, including immunohistochemistry and Transferase-mediated deoxyuridine triphosphate-biotin nick end labelling (TUNEL), was performed to confirm the role of sFRP1. Transwell chamber and angiogenesis assays were conducted to verify the effect of sFRP1 *in vitro*. We found that the control group showed negative radiological performance with successful implantation. Concurrently, the treated group showed visible lesions, a higher FDG uptake and increasing enhancement. The immunological and histological analysis confirmed the positive radiological performance with larger size, increasing proliferation, more microvessels and less apoptosis. The angiogenic up-regulation of sFRP1 overexpression were further verified with *in vitro* cell models. This preliminary study demonstrates that sFRP1 overexpression in gastric cancer cells leads to increased cell proliferation and angiogenesis, which may, in turn, contribute to positive PET/CT and CT performances.

Gastric cancer, with a five–year survival of only 20–30%, is the second leading cause of cancer modality in the world^1^,^2^,^3^. A good prognosis requires choosing the correct therapy and is closely correlated with tumour, node, metastasis (TNM) staging, histological classification as well as differentiation^4^,^5^. Currently, imaging has been widely used in assessments for the whole process of gastric cancer treatment. MDCT scanning has played an important role not only in the TNM staging of gastric cancers but also in the determination of tumour resectability^6^. However, radiological studies of patients with histologically proven gastric carcinoma have mainly been based on morphology. With the introduction of dual-energy spectral CT (DEsCT), the functional imaging aspect of CT has also been added to clinical applications, contributing to evaluations of therapeutic efficacy and predicting patient prognoses^7^,^8^,^9^. Based on their preference for aerobic glycolysis, F^18^-FDG, a glucose analog, has been exploited as a promising tracer in the diagnosis of malignancies, combined with positron emission tomography/computed tomography (PET/CT)^10^. Compared with CT, PET/CT shows improved accuracy for organ and distant lymph node metastases. Tumours larger in size, with deeper invasion, of an intestinal type, or at the gastroesophageal junction (GEJ) tend to evince a greater uptake of FDG^2^.

The increasing incidence and mortality have spurred researchers to identify its molecular mechanisms. Secreted frizzled-related protein 1 (sFRP1) contains a frizzled (FRZ)-type cysteine-rich domain (CRD), possessing 30–50% sequence similarity to those of Wnt receptor frizzled proteins. As the Wnt signalling pathway is a prerequisite for the process of embryo development, proliferation, differentiation and apoptosis in adult tissues, the dysregulated activation of the Wnt pathway may induce tumourigenesis^11^,^12^,^13^,^14^. Therefore, sFRP1 has been traditionally known as a Wnt antagonist by interacting with Wnt ligand^11^,^15^. Previous studies have shown the transcriptional silencing of sFRPs in various cancers. There was aberrant hypermethylation of the sFRP genes leading to sFRP1 inactivation in the early stages of gastric carcinogenesis^11^,^16^. However, emerging evidence suggests that sFRP1 may also promote tumour growth. Masaki *et al*. determined that some metastatic gastric cancers showed especially high expression of sFRP1, with the gene expression ratio between metastatic carcinoma cells: primary carcinoma cells >2.0^17^. Fifty-four percent of gastric patients in the previous study had highly expressed sFRP1, reflecting the controversial influence of sFRP1. In a previous study by our group, TGFβ signalling activation was considered to be the mechanism by which sFRP1 promotes the formation of tumours. sFRP1-overexpressing cells showed an increase in the expression of TGFβ1, its downstream targets, and TGFβ-mediated EMT^18^.

Studies on tumour imaging have focused on exploring the role of PET-CT in early response monitoring and seeking for the promising radiotracers, aiming at higher tumour-targeting efficacy and specificity based on mouse models^9^,^19^,^20^,^21^. There has been little research with respect to tumour imaging on the basis of altering tumour-related protein expression. Therefore, the combination of DEsCT and PET/CT was utilized in the current study, in order to assess tumours in terms of both morphology and function, reflecting tumour aggressiveness. In this study, we first explored imaging findings induced by the sFRP1-overexpressing gastric cancer cell *in vivo* using mouse models, with PET/CT and DEsCT. Further analysis, including immunohistochemistry, transferase-mediated deoxyuridine triphosphate-biotin nick end labelling (TUNEL), was performed to confirm the role of sFRP1. Then, transwell chamber and angiogenesis assays were conducted to verify the effect of sFRP1 *in vitro*.

## Results

To investigate the effect of sFRP1 up-regulation on tumour imaging, we exploited the SGC-7901/vector to establish a treated group and SGC-7901/sFRP1 cells to establish a control, including T-1 (the first mouse in the treated group, pulmonary metastasis), T-2 to T-7 (the other 6 mice in the treated group, peritoneal seeding), C-1 (the first mouse in the control group, pulmonary metastasis) and C-2 to C-7 (the other 6 mice in the control group, peritoneal seeding).

### Overexpression of sFRP1 increased PET/CT and spectral CT performance

**Figure 1:**
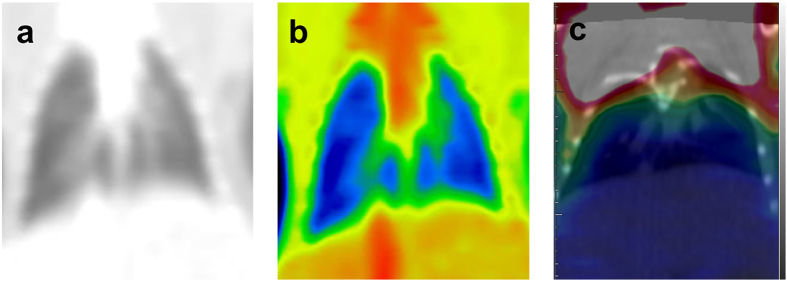
Tomogram of lung metastasis sample from spectral CT and PET/CT (C-1). (**a**) Coronal GSI monochromatic image of lungs obtained in the portal-dominant phase, (**b**) colour-overlay of the corresponding monochromatic image, (**c**) fused PET/CT image of the same lungs. Both sides of the lung field were clear in the GSI and PET/CT images. No visible lesion, obvious abnormal enhancement or higher FDG uptake was shown in the lungs. The corresponding SUV_max_ was 0.34.

**Figure 2:**
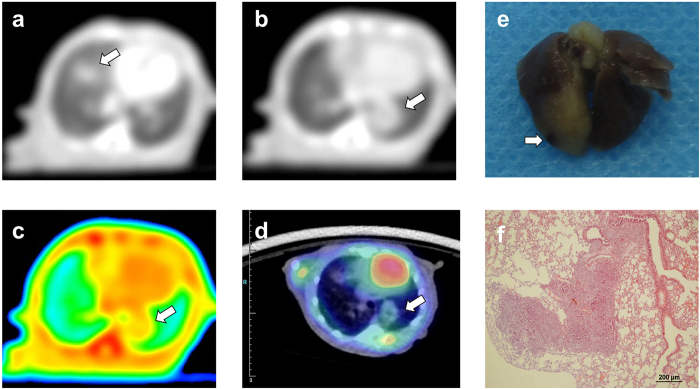
Tomogram of lung metastasis sample from spectral CT, PET/CT and corresponding HE pathology (T-1). (**a**–**c**) Axial GSI monochromatic images in portal phase: (**a**,**b**) lung window images on different slices, (**c**) color-scale monochromatic images. (**d**) transverse fused images of PET/CT, (**e**) gross specimen, (**f**) histological section. Transverse GSI images demonstrated small circular lesions in the bilateral lower lungs. (**a**,**b**) showed the maximum cross section of the two lesions, respectively. (**c**) further demonstrated the mild enhancement within the lesions. (**d**) depicted circular lesions with slight FDG uptake in the relevant location. The SUV_max_ values were 0.22 and 0.36, respectively. (**e**) demonstrated metastasis in right lower lung clearly, further certified by histological section (**f**). Arrows pointed out the metastatic nodules.

There were no obvious lesions, abnormal enhancement or high FDG uptake observed in the lungs of the C-1 mouse [Fig. 1]. The corresponding SUV_max_ was approximately 0.34. Two metastatic nodules in the bilateral lower lungs of T-1 were clearly revealed and were approximately 2.75 × 4.27 mm and 3.17 × 3.45 mm in diameter on PET-CT. Slight enhancement was visible in the lesions despite no obvious increased FDG uptake identified, with the SUV_max_ approximating 0.22 and 0.34 [Fig. 2].

**Figure 3:**
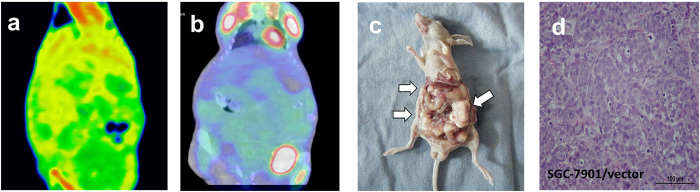
Tomogram of peritoneal metastasis sample from spectral CT, PET/CT and histological analysis (C-2). (**a**) GSI color-scale fused images obtained in portal phase, (**b**) fused PET/CT image, (**c**) gross specimen, (**d**) histological image. No visible lesion, obvious abnormal enhancement or high FDG uptake was shown in the abdominal cavity. The corresponding SUV_max_ was 0.39. (**c**) and (**d**) confirmed the successful implantation.

**Figure 4:**
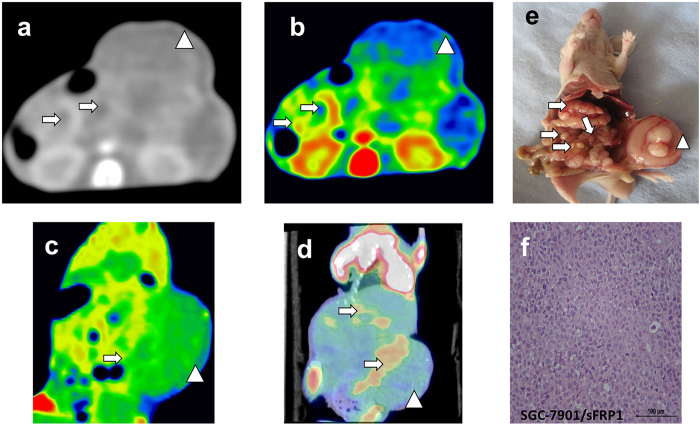
Tomogram of SGC-7901/sFRP1 induced peritoneal metastasis model from spectral CT, PET/CT and corresponding HE staining (T-2). (**a**–**c**) GSI monochromatic images in portal phase: (**a**) transverse monochromatic image, (**b**) transverse color-scale image, (**c**) coronal color-scale image. (**d**) coronal fused images of PET/CT, (**e**) gross specimen, (**f**) histological section. GSI images yielded excellent results of subcutaneous metastasis beyond the contour of abdomen, with peripheral enhancement confirmed by colour-scale image. PET/CT image depicted focal abnormal uptake of metastasis, including the peritoneal and subcutaneous ones. The corresponding SUV_max_ of subcutaneous and peritoneal metastasis were 1.2 and 0.98, respectively. (**e**,**f**) further illustrated a large number of peritoneal metastasis and huge subcutaneous metastasis shown on the GSI and PET/CT images. Arrows pointed out the peritoneal metastatic nodules, while arrow heads pointed out the subcutaneous metastasis.

Similarly, C-2 to C-7 [C3 shown in Supplementary Fig. 1] did not show any obvious positive performance with PET/CT and spectral CT [Fig. 3]. The SUV_max_ was approximately 0.3. However, radiological images of T-2 revealed one subcutaneous metastasis, which showed mild or moderate peripheral enhancement on DEsCT and much higher FDG uptake, with an SUV_max_ close to 1.2. The pathology-identified peritoneal tumours of T-2 were not differentiated from surrounding structures by DEsCT, whereas PET/CT coronal fused images depicted the focal abnormal uptake of metastases, with an SUV_max_ close to 0.98 [Fig. 4]. With successful peritoneal implantation, the results in the other mice (T-3 to T-7) were analogous to that of T-2, with SUV_max_ values of approximately 0.8, 1.06, 0.99, 0.83 and 0.96, respectively [T-3 to T-6 shown in Supplementary Figures 1,3,4 and 5]. A significant difference in the SUV_max_ of peritoneal metastasis was observed between the treated and control groups (P < 0.001).

### sFRP1 overexpression increased cell proliferation and angiogenesis

Gross specimen analysis demonstrated a nodule in C-1 that was 0.8 mm in diameter [Fig. 1]. Two metastatic nodules in the T-1 gross specimen were 4.1 and 3.2 mm in diameter, respectively, which was consistent with findings using PET-CT images (4.27 mm and 3.45 mm, respectively). The lesion in the inferior lobe of right lung is shown in Fig. 2, whereas the other lesion on the left side was not visible in this image. C-2 to C-7 showed four to ten nodules on the peritoneum, with 3.1 mm being the largest in size by gross specimen. The average size of peritoneal nodules from C-2 to C-7 was 1.13 mm (ranging from 0.5 mm to 3.1 mm). T-2 to T-7 revealed 8 to 21 peritoneal lesions with 6.11 mm as the largest size confirmed by gross specimen (mean 2.66 mm, ranging from 0.6 mm to 6.11 mm). Additionally, T-2 showed another subcutaneous lesion, with 13.6 mm as the largest size. No significant difference in tumour number was shown between the treated and control groups (P = 0.528). For the peritoneal tumour size, there was a significant difference between the treated and control groups (P < 0.001). Therefore, the treated group showed an increasing average size of tumours in C-3 and other positive radiological images in T-3 to T-6 [see supplementary results].

**Figure 5:**
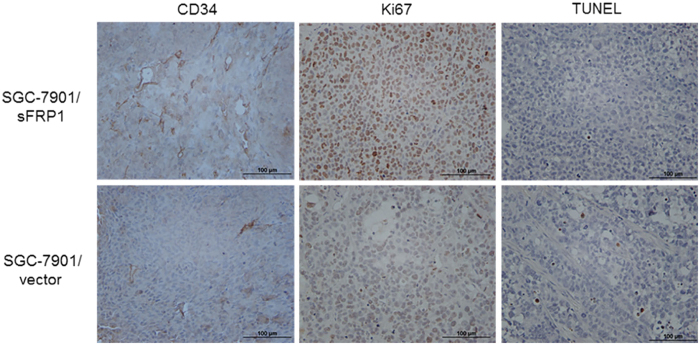
CD34, Ki67 and TUNEL assays staining in xenograft tumors. (**a**) *In vitro* angiogenesis assays were performed by treating HUVEC cells with culture supernatants of SGC-7901/vector or SGC-7901/sFRP1 cells. Representative pictures are shown (original magnification,x40). Numbers of tube formation per field are plotted. The data are shown as mean ± SD of three independent experiments (*p* < 0.05). (**b**) Migration of HUVECs was conducted using Boydon chamber assays. Same amount of SGC-7901/vector and SGC-7901/sFRP1 cells were plated in 24-well plate for 24 h, and the culture supernatants were collected and used as chemoattractants to HUVECs in the migration assays. Representative pictures are shown (left, original magnification,x100) and migrated cell numbers are plotted (right). The data are shown as mean ± SD of three independent experiments (*p* < 0.05).

**Figure 6:**
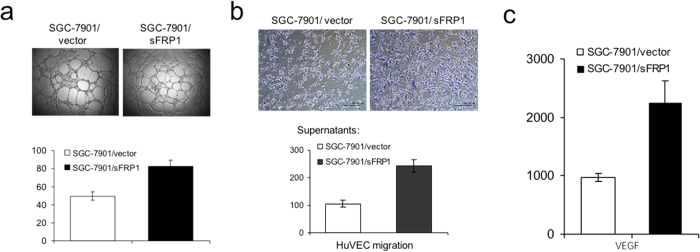
Overexpression of sFRP1 in SGC-7901 increased *In Vitro* angiogenesis. (**a**) *In vitro* angiogenesis assays were performed by treating HUVEC cells with culture supernatants of SGC-7901/vector or SGC-7901/sFRP1 cells. Representative pictures are shown (original magnification,x40). Number of tube formation per field are plotted. (**b**) Migration of HUVECs was conducted using Boydon chamber assays. Same amount of SGC-7901/vector and SGC-7901/sFRP1 cells were plated in 24-well plate for 24 h, and the culture supernatants were collected and used as chemoattractants to HUVECs in the migration assays. Representative pictures are shown (left, original magnification,x100) and migrated cell numbers are plotted (right). (**c**) VEGF levels in culture supernatants supernatants of SGC-7901/vector and SGC-7901/sFRP1 cells were examined using ELISA analysis.

After imaging, all tumours were dissected for further analysis. Histology and TUNEL were performed to measure apoptosis in the tumour tissue. TUNEL analysis showed that there were fewer apoptotic cells in tumours formed by sFRP1-overexpressing cells than by control cells [Fig. 5C]. Immunohistochemical staining revealed that tumours derived from sFRP1-overexpressing cells contained more proliferative cells (assayed by Ki-67 staining) and more microvessels (assayed by CD34 staining). We next tested whether the supernatant of sFRP1-overexpressing cell cultures promoted angiogenesis *in vitro*. We found that the supernatant of sFRP1-overexpressing cells indeed induced more tubular formation of HUVECs compared with control supernatant, demonstrating the angiogenic effect of sFRP1 *in vitro* [Fig. 6A]. Likewise, supernatant from sFRP1-overexpressing cells induced more HUVEC migration compared to supernatant from vector control cells. [Figure 6B]. sFRP1^22^ and ID1^23^ have been reported to increase tumour vessel density as well as the expression of vascular endothelial growth factor (VEGF), a well-known angiogenic factor induced by tumour growth factor β signalling^24^. Indeed, higher levels of VEGF were found in the culture supernatants of sFRP1-overexpressing cells compared to control cells [Fig. 6C, *left*].

We therefore demonstrated that the overexpression of sFRP1 in SGC-7901 increases tumourigenesis and induces positive performance by PET/CT and spectral CT.

## Discussion

Mice in the control group after successful implantation displayed no visible lesions, suspicious FDG uptake or enhancement. Compared with the control group, sFRP1 overexpression positively induced visibly larger nodules with increasing enhancement in lung metastasis and higher FDG uptake in peritoneal tumours. Simultaneously, subcutaneous metastases in the treated group showed positive functional performance both on DEsCT and PET/CT, i.e., increasing enhancement and FDG uptake, in addition to the visible size of the lesion. The SUV_max_ confirmed the increased FDG uptake. However, no obvious increased FDG uptake was identified in lung metastasis and no peritoneal tumours were differentiated in either control or treated groups by DEsCT. As verified by immunological and histological analysis, tumours from the treated group contained a greater number of proliferative cells, more microvessels and fewer apoptotic cells. Additionally, we demonstrated that sFRP1 contributed to *in vitro* angiogenesis and *in vivo* microvessel formation. Therefore, we argue that sFRP1 may contribute to positive performance in PET/CT and dual CT.

Great efforts have been put forth to show that FDG uptake is associated with tumour aggressiveness^25^. Progressive gastric carcinomas, represented as the depth of invasion, lymphatic permeation, vascular invasion and tumour size, showed higher FDG uptake^26^. In this study, the increasing number and larger size of tumours in the treated group demonstrated an increase in tumour aggressiveness. Consistently, the treated group with higher aggressiveness presented a positive performance, in contrast with the control group. In terms of quantitative evaluation, studies suggested that the SUV_max_ (one of the most popular candidates for semi-quantitative analysis of tumour glucose metabolism) has a positive correlation with proliferation in various malignancies^27^,^28^. Ki-67 was utilized as a quantitative biomarker for tumour aggressiveness, reflecting invasiveness and metastatic potential^29^,^30^. A significant moderate correlation coefficient was observed between SUV_max_ and the Ki-67 proliferation index (PI)^25^,^31^. The generation of a lethal tumour mass requires both tumour cell proliferation and angiogenesis^32^. Glucose metabolism was increased to supply sufficient energy for proliferation, leading to increased FDG accumulation in tumours with high growth rates, which explained the mechanism at cellular level^33^. In our study, the immunological analysis consistently demonstrated that sFRP1 overexpression resulted in increased cell proliferation and decreased apoptosis.

As described above, sFRP1-overexpressing cells showed an increase in the expression of TGFβ1, its downstream targets, and TGFβ-mediated EMT. TGFβ signalling activation may be the mechanism by which sFRP1 promotes tumourigenesis^18^. Previous research has also demonstrated the MCF-7 breast cancer cells cultured with TGFβ showed increased EMT, ^18^F-FDG uptake and glucose transporter (GLUT1) expression^34^. As a member of the multi-membrane-spanning facilitative transporter family, GLUT1 is considered to be the main functional transporter of glucose in most transformed cells^35^. Therefore, we hypothesized that TGFβ signalling activation promotes not only tumourigenesis but also GLUT1 expression and that both contribute to the observed positive performance in PET/CT and DEsCT.

Angiogenesis plays critical roles in the growth of cancer^36^. Angiogenesis is related to increased perfusion, blood volume and permeability, leading to increased contrast enhancement, which indicates that the development of new vessels within tumours determines their performance on contrast enhanced CT. Put another way, the intensity of neovascularization determines the contrast enhancement^37^. The degree of enhancement in the corticomedullary phase has been shown to correlate with microvessel density, in terms of renal parenchymal neoplasm^38^. A similar result was found in a study of pancreatic carcinoma in which the level of CT enhancement depended on the MVD number^39^. The enhancement of lung nodules appears to be an indicator of vascularity as well^40^. Consistent with these previous studies, a sFRP1 up-regulation model (T-1) showed increased numbers of microvessels assayed by CD34 staining and demonstrated visibly abnormal CT enhancement compared with a control group (C-1), which showed no abnormal enhancement.

In view of the spatial resolution of 64-slice CT, we considered the increased tumour size may partly contribute to visible nodules with CT, especially for nodules in T-1 (4.27 mm and 3.45 mm for the average size, respectively), which were much larger than C-1 (0.8 mm). As a result, a subcutaneous tumour with a much larger size (13.6 mm) was visible and presented peripheral enhancement, compared to other invisible peritoneal tumours (1.6 mm for the average size) in T2 by DEsCT. With limited resolution, peritoneal tumours with insufficiently increasing size for visibility showed similar enhancement with the surrounding structures on DEsCT, which may contribute to the negative performance observed in T2 to T7. Tumour cell proliferation and angiogenesis were both considered to be necessary in the development of a detectable tumour^32^. Nevertheless, PET-CT provides functional imaging for tumours rather than mere morphology. Peritoneal nodules showed positive performance with higher SUV_max_ on PET/CT. The reason why the visible lung metastases from T-1, with a larger size, showed similar FDG uptake to those from C-1 in this study is currently unclear. We believe that size was not the determinant that changed the imaging appearance of tumours.

In this study, we explored imaging findings induced by sFRP1-overexpressing gastric cancer cells *in vivo* using mouse models with PET/CT and DEsCT. Additionally, we demonstrated that sFRP1 overexpression in gastric cancer induces positive performance in imaging modalities. Nevertheless, there are several limitations to our study. First, GLUT1 expression was not measured in this study. Second, the sample size used in our study was relatively small. More quantitative studies should be carried out to better understand this relationship. Future studies should focus on how sFRP1 promotes endothelial cell migration and invasion.

In conclusion, sFRP1 overexpression in gastric cancer cells leads to more angiogenesis and increasing proliferation, which may, in turn, induce positive PET/CT and CT performance.

## Methods

### Ethics statement

This study was performed in strict accordance with the standards established by the Guidelines for the Care and Use of Laboratory Animals of Shanghai Jiao Tong University and was approved by the laboratory Animal Ethics Committee of Ruijin Hospital (Permit Number: 112). Mice were anaesthetized with an intraperitoneal injection of sodium pentobarbital (50 mg/kg) anaesthesia to minimize suffering.

### Cell lines and cell transfection

The moderately differentiated SGC-7901 human gastric cancer cell line was purchased from the Chinese Academy of Sciences in Shanghai. Cells were propagated in RPMI-1640 supplemented with 10% foetal bovine serum, 100 units/ml streptomycin, and 100 μg/ml penicillin, at 37 °C under 5% CO_2_. SFRP1-overexpressing models were generated with the SGC-7901 cell line, which normally expresses low or undetectable levels of sFRP1.

Plasmid containing Myc-DDK-tagged ORF clone of Homo sFRP1 was purchased from Origene (Origene Technologies, Rockville, MD). Transfection was carried out using Lipofectamine^®^ 2000 (Invitrogen, Carlsbad, CA) in accordance with the manufacturer’s instructions.

### Animal model

Six-week-old male athymic BALB/c (nu/nu) mice, ranging in weight from 25 g to 30 g, were obtained from the Animal Center (CAS, Shanghai, China) and housed under specific pathogen-free conditions in animal facility. The mice were randomly assigned to two groups, i.e., the treated group and control group. Then, 100-μl (1 × 10^6 ^cells/ml) suspensions of SGC-7901/sFRP1 cells were administered via the tail vein and 150-μl (2 × 10^6 ^cells/ml) suspensions were administered via the abdominal cavity to establish xenograft models of pulmonary metastasis and peritoneal seeding, respectively. Similarly, SGC-7901/vector cells were administered to establish the control group. After a period of 28 days of growth, tumour imaging and analysis were performed.

### Tumour imaging

#### PET/CT SCAN

^18^F-FDG PET/CT scan was performed on a micro-PET/CT (Inveon mPET/CT; Siemens Preclinical Solution, Knoxville, Tennessee). The scope of scanning was from the head to the tail. Mice were fasted for 12 hours before ^18^F-FDG PET scans but allowed free access to water. They were imaged in a supine position after general anaesthesia, and the CT component was performed before the emission component. The scanning parameters were a tube voltage of 80 kV and a tube current of 0.5 mA (with PET images taken at the same scope). Sixty minutes after an i.v. injection of 7.4 MBq of ^18^F-FDG, PET scans were acquired. The maximum standardized uptake value (SUV_max_) was calculated as the semi-quantitative analysis indicator. All PET images were corrected for emission scatter and attenuation.

#### DEsCT SCAN

The mice also underwent spectral CT imaging on a high-definition CT scanner (Discovery CT750HD, GE Healthcare, Milwaukee, WI) using the dual-energy spectral imaging mode with a single tube, fast kilovoltage switching between 80 kVp and 140 kVp in less than 0.5 ms. The other parameters included a tube current of 600 mA, a rotation speed (temporal resolution) of 0.5 s, a helical pitch of 0.531, 40-mm detector coverage, a collimation thickness of 0.625 mm, a 32-cm field of view (FOV), and a 512 × 512 reconstruction matrix. Mice were injected with the non-ionic contrast medium iopamidol (30 g of iodine per 100 ml, Shanghai) with a power injector (Ulrich REF XD 2060-Touch, Germany) at a rate of 0.5 ml/s and a dosage of 0.1 ml/100 g through the tail vein. The dual phase scans began at 7 s (arterial phase), 15 s (portal venous phase), and 25 s (delayed phase), after the injection of the contrast medium^40^. The image reconstruction thickness was 0.625 mm at an interval of 0.625 mm. Images were reconstructed with a 25-cm display field of view (DFOV) and a 512 × 512 reconstruction matrix size. Three types of images were reconstructed from the single dual-energy spectral CT acquisition for analysis: a set of polychromatic images corresponding to the conventional 140-kVp imaging, water- and iodine-based material-decomposition images, and 101 sets of monochromatic images corresponding to photon energies ranging from 40 to 140 keV. The spectral CT images were analysed with the Gemstone Spectral Imaging (GSI) Viewer software (GE Healthcare, Waukesha, Wisconsin), with a standard soft-tissue display window preset (WL 40 and WW 400).

From the monochromatic and material decomposition image sets, cross-section, multi-planar reformat (MPR) and colour–scaled images were obtained. Two radiologists conducted the analysis. All the lesions were confirmed on monochromatic images of DEsCT, combined with the corresponding iodine-based material decomposition data. Metabolic information was further evaluated on PET/CT images. We then compared results from two modalities between 2 groups.

#### Immunological and histological analysis of tumours in xenografts

Tumours were then harvested and processed for immunohistochemistry. Consecutive tissue sections were hydrated for antigen retrieval carried out in citrate buffer (pH 6.0) at 95 °C. After peroxidase blocking, sections were incubated with Ki67 and CD34 rabbit polyclonal antibodies (Santa Cruz biotechnology Inc., Santa Cruz, CA) followed by HRP–conjugated secondary antibodies (Dako Cytomation, Carpinteria, CA). For quantification of Ki67, the percentage of positive cells was scored in five fields per slide. Microvessels were counted as CD34–positive endothelial cells in five fields per slide under microscopy.

#### TUNEL assays

The TUNEL assays were performed using a FragEL™ DNA Fragmentation Detection kit (Calbiochem, La Jolla, CA) in accordance with the manufacturer’s instructions. The negative control was generated by substituting H_2_O for the TdT in the reaction mixture during the labelling step. The positive control was generated by covering the entire specimen with 1 μg/μl of DNase I in 1 × TBS/1 mM MgSO_4_ following proteinase K treatment for 20 min. All other steps were performed as previously described.

#### *In vitro* angiogenesis assays

Angiogenesis assays were performed according to the manufacturer’s instructions. Briefly, 50 μL of ECMatrix^TM^ solution was transferred to each well of a precooled 96-well tissue culture plate on ice. After incubation at 37 °C for 1 hour to allow the matrix solution to solidify, human umbilical vein endothelial cells (HUVECs) were harvested, resuspended and seeded at 5 × 10^3^ cells per well onto the surface of the polymerized ECMatrix^TM^. Cells were incubated with conditioned medium from SGC-7901/vector and SGC-7901/sFRP1 cells at 37 °C for 12 hours. The ability to form tubes was evaluated by counting the tubular number, the tubular length and tubular intersecting nods in five random fields.

#### Endothelial cell migration assays

Cell migration was analysed by a transwell chamber assay. Ten percent FCS or condition medium was used as the chemoattractant. Endothelial cells migrated through the transwell membrane and those that attached onto the lower surface of the insert were fixed and stained followed by counting under a light microscope.

### Statistical analysis

Values represented the mean ± standard deviation (SD) of samples measured in triplicate. Each experiment was repeated three times. Student’s t-test and two-tailed distribution were conducted on all quantitative data. A *P*-value < 0.05 was taken as the level of significance.

## Additional Information

**How to cite this article:** Lin, H. *et al*. Secreted frizzled-related protein 1 overexpression in gastric cancer: Relationship with radiological findings of dual energy spectral CT and PET-CT. *Sci. Rep.* **7**, 42020; doi: 10.1038/srep42020 (2017).

**Publisher's note:** Springer Nature remains neutral with regard to jurisdictional claims in published maps and institutional affiliations.

## Supplementary Material

Supplementary Figures

## References

[b1] Pisani P., Parkin D. M., Bray F. & Ferlay J. Estimates of the worldwide mortality from 25 cancers in 1990. International journal of cancer 83, 18–29 (1999). 10602059 10.1002/(sici)1097-0215(19991210)83:6<870::aid-ijc35>3.0.co;2-9

[b2] Wu C. X. & Zhu Z. H. Diagnosis and evaluation of gastric cancer by positron emission tomography. World journal of gastroenterology 20, 4574–4585, doi: 10.3748/wjg.v20.i16.4574 (2014). 24782610 PMC4000494

[b3] Tenderenda M., Rutkowski P., Jesionek-Kupnicka D. & Kubiak R. Expression of CD34 in gastric cancer and its correlation with histology, stage, proliferation activity, p53 expression and apoptotic index. Pathol Oncol Res 7, 129–134 (2001). 11458276 10.1007/BF03032579

[b4] Ahn M. J. . Clinical prognostic values of vascular endothelial growth factor, microvessel density, and p53 expression in esophageal carcinomas. J Korean Med Sci 17, 201–207, doi: 10.3346/jkms.2002.17.2.201 (2002). 11961303 PMC3054851

[b5] Pan Z. . Determining gastric cancer resectability by dynamic MDCT. Eur Radiol 20, 613–620, doi: 10.1007/s00330-009-1576-2 (2010). 19707768

[b6] Fang N. . Clinicopathological characteristics and prognosis of gastric cancer with malignant ascites. Tumour biology: the journal of the International Society for Oncodevelopmental Biology and Medicine 35, 3261–3268, doi: 10.1007/s13277-013-1426-3 (2014). 24282088

[b7] Kim M. J., Hong J. H., Park E. S. & Byun J. H. Gastric metastasis from primary lung adenocarcinoma mimicking primary gastric cancer. World journal of gastrointestinal oncology 7, 12–16, doi: 10.4251/wjgo.v7.i3.12 (2015). 25780510 PMC4357873

[b8] Maeda H., Kobayashi M. & Sakamoto J. Evaluation and treatment of malignant ascites secondary to gastric cancer. World journal of gastroenterology 21, 10936–10947, doi: 10.3748/wjg.v21.i39.10936 (2015). 26494952 PMC4607895

[b9] Bensinger S. J. & Christofk H. R. New aspects of the Warburg effect in cancer cell biology. Semin Cell Dev Biol 23, 352–361, doi: 10.1016/j.semcdb.2012.02.003 (2012). 22406683

[b10] Zhao C. H., Bu X. M. & Zhang N. Hypermethylation and aberrant expression of Wnt antagonist secreted frizzled-related protein 1 in gastric cancer. World J Gastroenterol 13, 2214–2217 (2007). 17465504 10.3748/wjg.v13.i15.2214 PMC4146847

[b11] Gu W., Li X. & Wang J. miR-139 regulates the proliferation and invasion of hepatocellular carcinoma through the WNT/TCF-4 pathway. Oncol Rep 31, 397–404, doi: 10.3892/or.2013.2831 (2014). 24190507

[b12] Shi Y., He B., You L. & Jablons D. M. Roles of secreted frizzled-related proteins in cancer. Acta Pharmacol Sin 28, 1499–1504, doi: 10.1111/j.1745-7254.2007.00692.x (2007). 17723183

[b13] Amin N. & Vincan E. The Wnt signaling pathways and cell adhesion. Front Biosci (Landmark Ed.) 17, 784–804 (2012). 10.2741/3957 22201774

[b14] Jones S. E. & Jomary C. Secreted Frizzled-related proteins: searching for relationships and patterns. Bioessays 24, 811–820, doi: 10.1002/bies.10136 (2002). 12210517

[b15] Sugai T. . Molecular analysis of gastric differentiated-type intramucosal and submucosal cancers. Int J Cancer 127, 2500–2509, doi: 10.1002/ijc.25271 (2010). 20178104

[b16] Mori M. . Analysis of the gene-expression profile regarding the progression of human gastric carcinoma. Surgery 131, S39–47 (2002). 11821786 10.1067/msy.2002.119292

[b17] Qu Y. . High levels of secreted frizzled-related protein 1 correlate with poor prognosis and promote tumourigenesis in gastric cancer. European journal of cancer 49, 3718–3728, doi: 10.1016/j.ejca.2013.07.011 (2013). 23927957

[b18] Perk L. R. . Quantitative PET imaging of Met-expressing human cancer xenografts with 89Zr-labelled monoclonal antibody DN30. Eur J Nucl Med Mol Imaging 35, 1857–1867, doi: 10.1007/s00259-008-0774-5 (2008). 18491091

[b19] Wiehr S. . Preclinical evaluation of a novel c-Met inhibitor in a gastric cancer xenograft model using small animal PET. Mol Imaging Biol 15, 203–211, doi: 10.1007/s11307-012-0580-0 (2013). 22864665

[b20] Xu B. . Evaluation of 68Ga-labeled MG7 antibody: a targeted probe for PET/CT imaging of gastric cancer. Sci Rep 5, 8626, doi: 10.1038/srep08626 (2015). 25733152 PMC4346831

[b21] Dufourcq P. . FrzA, a secreted frizzled related protein, induced angiogenic response. Circulation 106, 3097–3103 (2002). 12473558 10.1161/01.cir.0000039342.85015.5c

[b22] Ling M. T. . Overexpression of Id-1 in prostate cancer cells promotes angiogenesis through the activation of vascular endothelial growth factor (VEGF). Carcinogenesis 26, 1668–1676, doi: 10.1093/carcin/bgi128 (2005). 15905202

[b23] Saito H. . The expression of transforming growth factor-beta1 is significantly correlated with the expression of vascular endothelial growth factor and poor prognosis of patients with advanced gastric carcinoma. Cancer 86, 1455–1462 (1999). 10526273 10.1002/(sici)1097-0142(19991015)86:8<1455::aid-cncr11>3.0.co;2-l

[b24] Hu S. L. . Role of SUV(max) obtained by 18F-FDG PET/CT in patients with a solitary pancreatic lesion: predicting malignant potential and proliferation. Nuclear medicine communications 34, 533–539, doi: 10.1097/MNM.0b013e328360668a (2013). 23503000

[b25] Yamada A., Oguchi K., Fukushima M., Imai Y. & Kadoya M. Evaluation of 2-deoxy-2-[18F]fluoro-D-glucose positron emission tomography in gastric carcinoma: relation to histological subtypes, depth of tumor invasion, and glucose transporter-1 expression. Ann Nucl Med 20, 597–604 (2006). 17294670 10.1007/BF02984657

[b26] Wu X. . Glucose metabolism correlated with cellular proliferation in diffuse large B-cell lymphoma. Leuk Lymphoma 53, 400–405, doi: 10.3109/10428194.2011.622420 (2012). 21913807

[b27] Tchou J. . Degree of tumor FDG uptake correlates with proliferation index in triple negative breast cancer. Mol Imaging Biol 12, 657–662, doi: 10.1007/s11307-009-0294-0 (2010). 20012701

[b28] Oka S., Uramoto H., Shimokawa H., Iwanami T. & Tanaka F. The expression of Ki-67, but not proliferating cell nuclear antigen, predicts poor disease free survival in patients with adenocarcinoma of the lung. Anticancer Res 31, 4277–4282 (2011). 22199292

[b29] Dong M. . Possible prognostic significance of p53, cyclin D1 and Ki-67 in the second primary malignancy of patients with double primary malignancies. Int J Clin Exp Pathol 7, 3975–3983 (2014). 25120774 PMC4129009

[b30] Watanabe R. . SUVmax in FDG-PET at the biopsy site correlates with the proliferation potential of tumor cells in non-Hodgkin lymphoma. Leukemia & lymphoma 51, 279–283, doi: 10.3109/10428190903440953 (2010). 20038236

[b31] Folkman J. Angiogenesis. Annu Rev Med 57, 1–18, doi: 10.1146/annurev.med.57.121304.131306 (2006). 16409133

[b32] Kurokawa T. . Expression of GLUT-1 glucose transfer, cellular proliferation activity and grade of tumor correlate with [F-18]-fluorodeoxyglucose uptake by positron emission tomography in epithelial tumors of the ovary. Int J Cancer 109, 926–932, doi: 10.1002/ijc.20057 (2004). 15027127

[b33] Li W. . Increased 18F-FDG uptake and expression of Glut1 in the EMT transformed breast cancer cells induced by TGF-beta. Neoplasma 57, 234–240 (2010). 20353274 10.4149/neo_2010_03_234

[b34] Mueckler M. Facilitative glucose transporters. Eur J Biochem 219, 713–725 (1994). 8112322 10.1111/j.1432-1033.1994.tb18550.x

[b35] Folkman J. Tumor angiogenesis. Adv Cancer Res 43, 175–203 (1985). 2581424 10.1016/s0065-230x(08)60946-x

[b36] Miles K. A. Tumour angiogenesis and its relation to contrast enhancement on computed tomography: a review. Eur J Radiol 30, 198–205 (1999). 10452718 10.1016/s0720-048x(99)00012-1

[b37] Jinzaki M. . Double-phase helical CT of small renal parenchymal neoplasms: correlation with pathologic findings and tumor angiogenesis. J Comput Assist Tomogr 24, 835–842 (2000). 11105696 10.1097/00004728-200011000-00002

[b38] Wang Z. Q. . Correlation of CT enhancement, tumor angiogenesis and pathologic grading of pancreatic carcinoma. World J Gastroenterol 9, 2100–2104 (2003). 12970915 10.3748/wjg.v9.i9.2100 PMC4656683

[b39] Swensen S. J., Brown L. R., Colby T. V., Weaver A. L. & Midthun D. E. Lung nodule enhancement at CT: prospective findings. Radiology 201, 447–455, doi: 10.1148/radiology.201.2.8888239 (1996). 8888239

[b40] Ma G. . Assessment of hemodynamics in a rat model of liver cirrhosis with precancerous lesions using multislice spiral CT perfusion imaging. Biomed Res Int 2013, 813174, doi: 10.1155/2013/813174 (2013). 23865067 PMC3705863

