# Supplementary material for: Secreted frizzled-related protein 1 overexpression in gastric cancer: Relationship with radiological findings of dual-energy spectral CT and PET-CT
Source: Sci Rep. 2017 Feb 7;7:42020. doi: 10.1038/srep42020 (PMC5294577; doi:10.1038/srep42020)
Supplement: Supplementary Figures [file srep42020-s1.doc]

# Secreted frizzled-related protein 1 overexpression in gastric cancer: Relationship with radiological findings of dual energy spectral CT and PET-CT

Huimin Lin^a^, Guoyuan Yang^b^, Bei Ding^a^, Miao Zhang^c^, Mingjun Zhang^d^, Fuhua Yan^a^, Ying Qu^e,*^, Huan Zhang^a,*^

^a^Department of Radiology, Ruijin Hospital, Shanghai Jiaotong University School of Medicine，Shanghai 200025, People’s Republic of China

^b^Department of Neurology, Ruijin Hospital, Shanghai Jiaotong University School of Medicine, Shanghai 200025, People’s Republic of China

^c^Department of Nuclear Medicine, Ruijin Hospital, Shanghai Jiaotong University School of Medicine，Shanghai 200025, People’s Republic of China

^d^Laboratory Animal Research Center，Ruijin Hospital, Shanghai Jiaotong University School of Medicine，Shanghai 200025, People’s Republic of China

^e^Cedars-Sinai medical center, 8700 beverly Blvd, Los Angeles, Ca90048, USA

*Email: huanzhangy@126.com; Ying.Qu@cshs.org

**Supplementary Information**


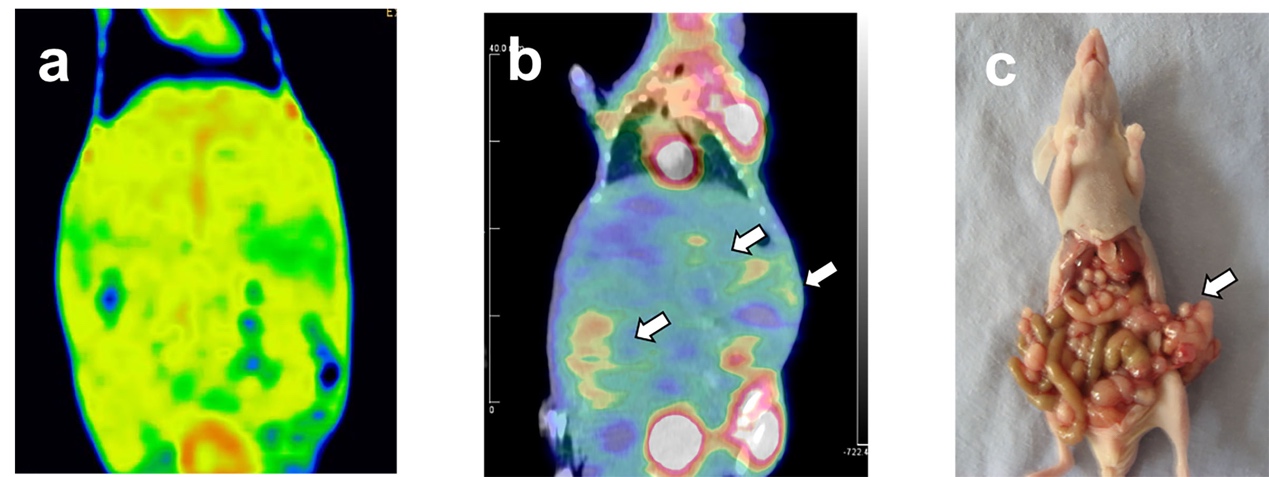


**Figure 1．****Tomogram of SGC-7901 /sFRP1 induced peritoneal metastasis model by DEsCT and PET/CT and corresponding gross specimen(T-3).**

**(a，b)** The coronal fused images of DEsCT and PET/CT depicted focal abnormal uptake of metastases. The corresponding SUV_max_ was about 0.8. **(c)** Gross specimen illustrated 21 nodules of peritoneal metastases. Arrows pointed out the metastatic nodules.


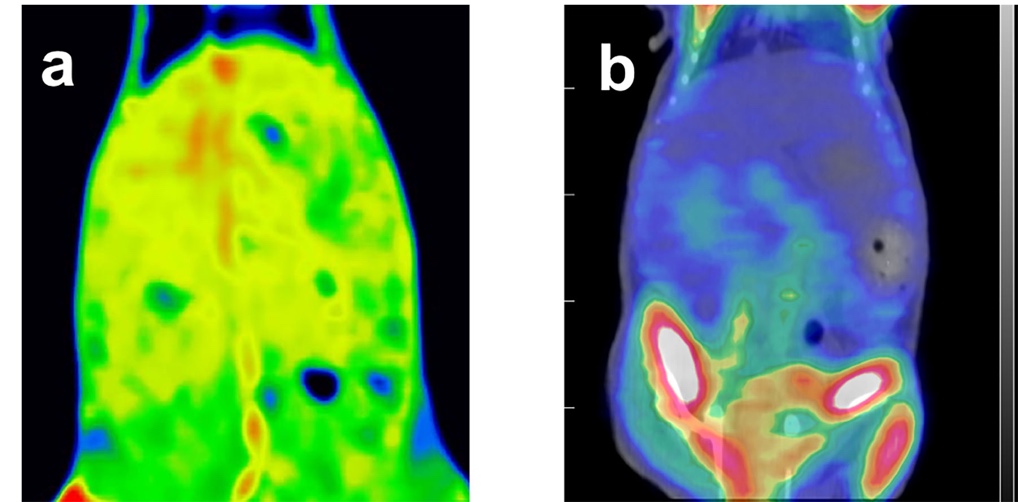


**Figure 2．Tomogram of SGC-7901 /vector induced peritoneal metastasis model by DEsCT and PET/CT (C-3).**

**(a，b)** No visible lesion, obvious abnormal enhancement or high FDG uptake was shown in the coronal fused images by spectral CT and PET/CT. The corresponding SUVmax was approximately 0.30.


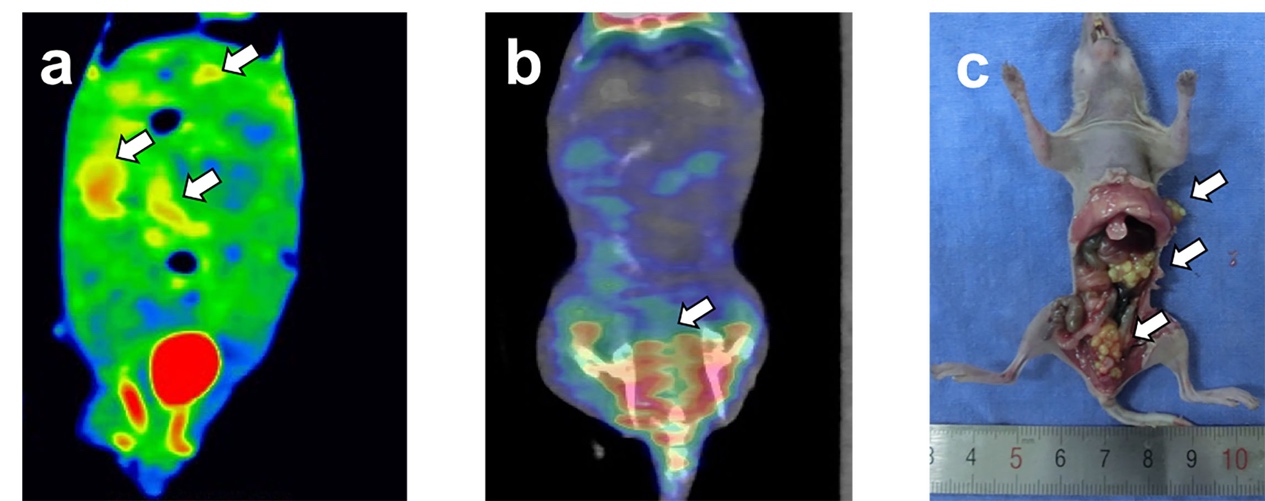


**Figure 3. Tomogram of SGC-7901 /sFRP1 induced peritoneal metastasis model by DEsCT and PET/CT and corresponding gross specimen(T-4).**

**(a，b)** The coronal fused images of DEsCT and PET/CT depicted focal abnormal uptake of metastases on two different slice. The corresponding SUVmax was approximately 1.06. **(c)** Gross specimen illustrated 22 nodules of peritoneal metastases. Arrows pointed out the metastatic nodules corresponding to the radiographic finding.


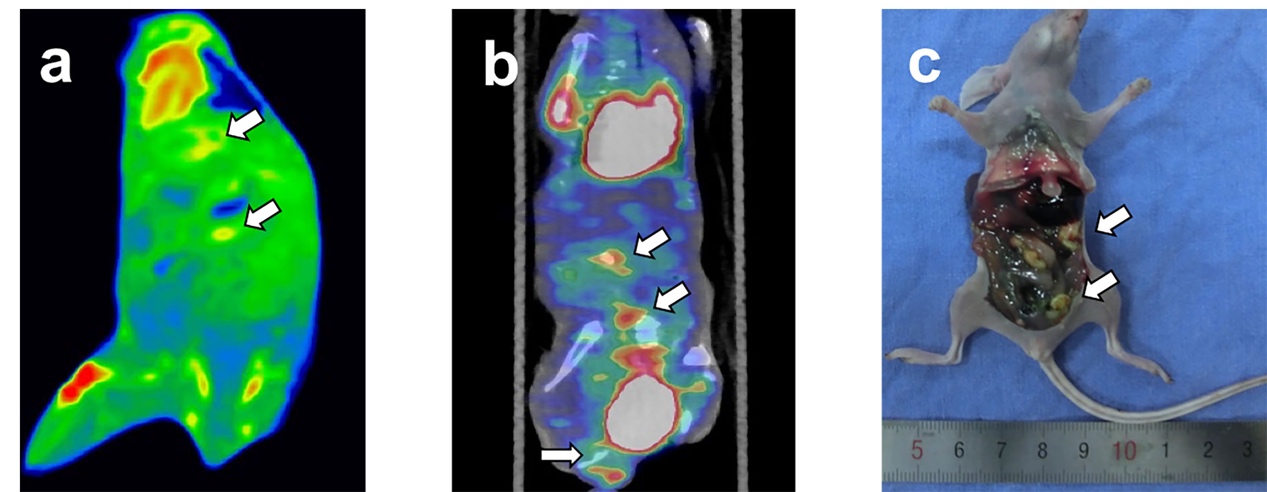


**Figure 4. Tomogram of SGC-7901 /sFRP1 induced peritoneal metastasis model by DEsCT and PET/CT and corresponding HE pathology(T-5).**

**(a，b)** The coronal fused images of DEsCT and PET/CT depicted focal abnormal uptake of metastases. The corresponding SUVmax was approximately 0.99. **(c)** Gross specimen illustrated 25 nodules of peritoneal metastases. Arrows pointed out the metastatic nodules.


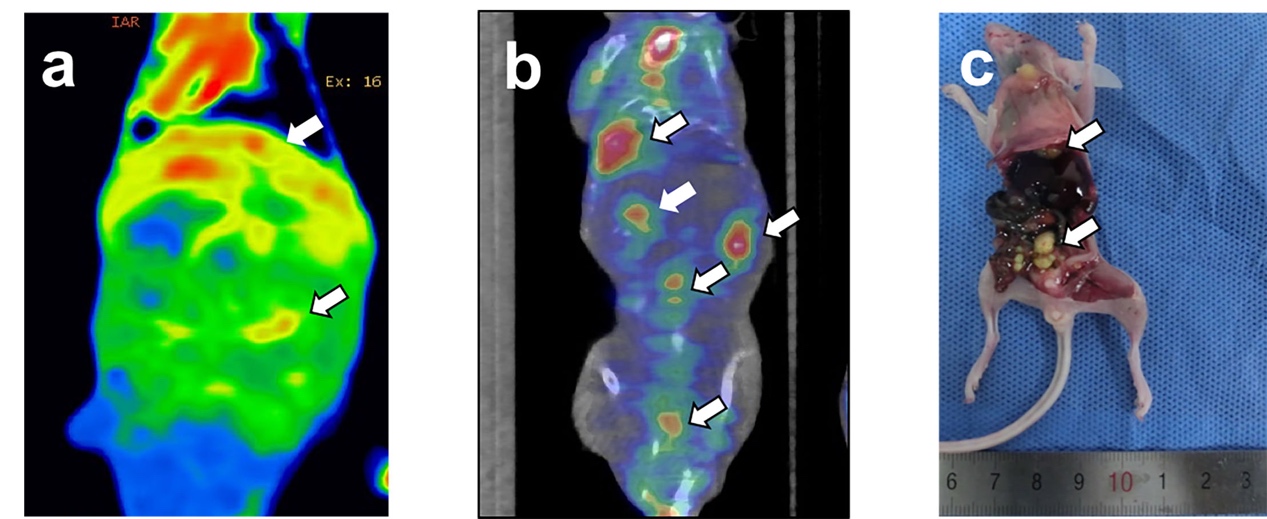


**Figure 5. Tomogram of SGC-7901 /sFRP1 induced peritoneal metastasis model by DEsCT and PET/CT and corresponding HE pathology(T-6).**

**(a，b)** The coronal fused images of DEsCT and PET/CT depicted focal abnormal uptake of metastases. The corresponding SUV_max_ value was approximately 0.83. **(c)** Gross specimen illustrated 30 nodules of peritoneal metastases, including the right subphrenic lesions. Arrows pointed out the metastatic nodules.
